# Intravenous thrombolysis and risk of early-onset post-stroke depression: a propensity score matched cohort study

**DOI:** 10.3389/fneur.2024.1385266

**Published:** 2024-11-06

**Authors:** Jieyi Lu, Lulu Zhang, Yi Zhang, Huawu Mao, Qi Fang

**Affiliations:** ^1^Department of Neurology, The First Affiliated Hospital of Soochow University, Suzhou, China; ^2^Department of Neurology, The Fourth Affiliated Hospital of Soochow University (Dushu Lake Hospital), Suzhou, China; ^3^Department of Neurology, The Affiliated Changzhou No. 2 People’s Hospital of Nanjing Medical University, Changzhou, China; ^4^Department of Neurology, The Affiliated Taizhou Second People’s Hospital of Yangzhou University, Taizhou, China

**Keywords:** intravenous thrombolysis, post-stoke depression, propensity score method, inverse probability of treatment weighting, pairwise algorithmic

## Abstract

**Background:**

Depression is common in stroke survivors and affects their recovery and quality of life (QoL). Intravenous thrombolysis (IVT) can improve post-stroke outcomes but the impact on early-onset post-stroke depression (PSD) is unclear. This was evaluated by comparing depression symptoms between patients with acute ischemic stroke (AIS) with vs. without IVT.

**Methods:**

This retrospective cohort study included 633 patients with AIS. The 17-item Hamilton Depression Rating Scale was used to evaluate depression in patients 14–21 days after stroke onset. Propensity score matching was used to minimize intervention bias between the two groups.

**Results:**

Of the 633 patients, 120 (19.0%) received IVT and 513 (81%) did not. Before matching, the prevalence of early-onset PSD was lower in the IVT group than in the non-IVT group (18.3% vs. 29.2%, *p* < 0.05). In the multivariate logistic regression analysis, the risk of early-onset PSD was significantly lower in the IVT group than in the non-IVT group [odds ratio (OR) = 0.48; 95% confidence interval: 0.28–0.83]. The results were stable after adjusting for potential confounders by inverse probability of treatment weighting and using a pairwise algorithm based on propensity scores (ORs between 0.44 and 0.61, all *p* < 0.05); were robust to unmeasured confounding as assessed by *E*-value analysis; and were consistent in subgroup analyses.

**Conclusion:**

IVT is associated with a reduced risk of early-onset PSD and can improve the QoL of patients with AIS during post-stroke recovery.

## 1 Introduction

Post-stroke depression (PSD) is a common occurrence during recovery from acute ischemic stroke (AIS), with a prevalence ranging from 29 to 43% (1, 2, 3, 4, 5). PSD negatively impacts neurologic recovery, quality of life (QoL), and morality risk in stroke survivors (6). PSD manifests as a loss of interest in or enjoyment of life (7), fatigue (8), sleep disturbance (9), difficulty concentrating, and lack of motivation (10). The etiology of PSD is complex; risk factors include pre-stroke factors such as female sex (11) and history of depression (12), stroke-related factors such as stroke severity and location of cerebral infraction (13), and post-stroke factors such as degree of inflammation (14) and lack of social support (15).

Intravenous thrombolysis (IVT) with the recombinant tissue plasminogen activator (rtPA) alteplase is an approved systemic reperfusion therapy for patients with AIS (16) that improves functional outcomes and enhances QoL by salvaging the ischemic penumbra (17). There are limited data on the potential effects of IVT on PSD. One prospective cohort study found that the frequency of depression was similar between stroke survivors who received thrombolysis and those who did not, although the authors concluded that thrombolysis therapy nonetheless had a positive—albeit indirect—effect on patients’ mood following stroke (18). In the WAKE-UP trial, rates of depression at 90 days were lower in patients with IVT after AIS than in those without IVT, which was only partly explained by a reduction in functional disability (19). Given that the restoration of function after stroke can influence the occurrence of PSD (20), the early effect of IVT on PSD warrants investigation. Studies to date have focused on longer-term outcomes (3 months or 1 year). The present study examined the relationship between IVT and early-onset PSD in the early stages after stroke when neurologic function has not stabilized or recovered.

## 2 Materials and methods

### 2.1 Data source

This retrospective study used data from the stroke neuropsychology patient database of the First Affiliated Hospital of Soochow University, which contains information on AIS patients who received care in the stroke unit. The study was designed according to the tenets of the Declaration of Helsinki and was approved by the Institutional Review Board (IRB no. 2023–397). Results are reported according to Strengthening the Reporting of Observational Studies in Epidemiology (STROBE) guidelines (21). All participants included in the study provided written, informed consent.

### 2.2 Study design and population

**Figure 1:**
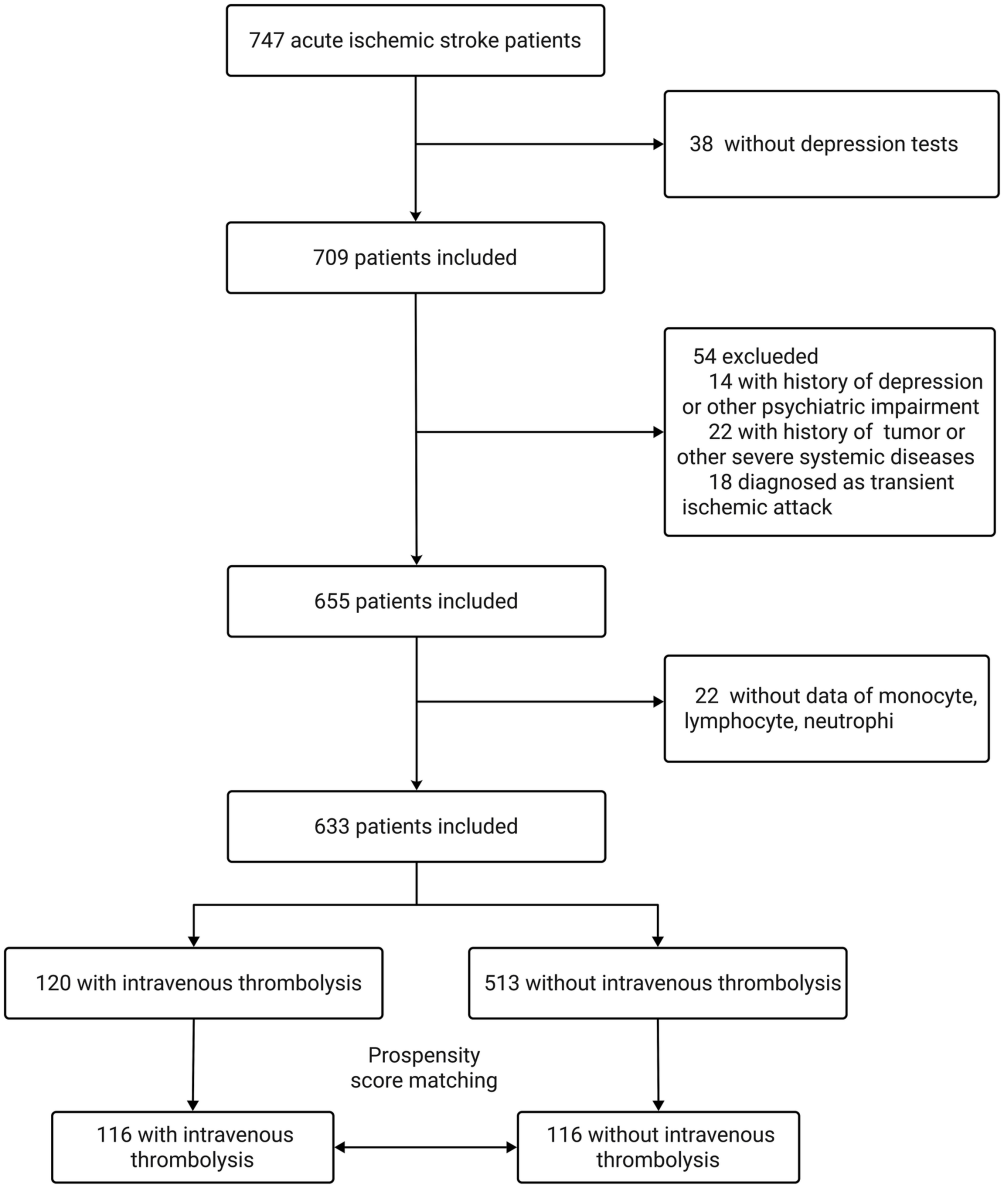
Participant inclusion flowchart.

A total of 747 consecutive patients with AIS (within 3 days of symptom onset) admitted to the First Hospital of Soochow University between March 2018 and February 2023 were screened. Inclusion criteria were as follows: aged 18–85 years, admitted within 3 days after onset of AIS (primary or recurrent stroke), and willing and able to complete the psychological evaluation. Exclusion criteria were severe aphasia, apoplexy, or unconsciousness hindering the completion of depression tests (*n* = 38); history of depression, anxiety, and other psychiatric impairment (*n* = 14); history of tumor or severe systemic disease (*n* = 22); and diagnosis of transient ischemic attack (*n* = 18). Another 22 patients were excluded for missing laboratory data (e.g., monocyte, lymphocyte, and neutrophil counts). Ultimately, 633 patients were enrolled in the study. After propensity score matching (PSM) between patients who received IVT and those who did not on select baseline characteristics, there were 116 patients in each group (Figure 1).

### 2.3 Study exposure

The primary independent variable was receipt of IVT within 4.5 h of stroke onset. The standard dose was 0.9 mg/kg and total dose was <90 mg. The first dose was 10% of the total dose, which was administered intravenously over 1 min; the remaining 90% of the dose was continuously delivered intravenously over 1 h using a microinjection pump according to 2021 European guidelines (16).

### 2.4 Diagnosis of early-onset PSD

The primary outcome of the study was a diagnosis of depression in the acute stage after ischemic stroke. Early-onset PSD was evaluated with the 17-item Hamilton Depression Scale (HAMD17) 14–21 days after stroke onset (22) using the established threshold score of ≥7 (23).

### 2.5 Clinical data

Fasting blood samples were collected on the morning after admission and demographic characteristics and risk factors were recorded within 6 h after admission.

Clinical parameters known to influence the occurrence of PSD were assessed, including demographic characteristics such as age and sex as well as vascular risk factors such as history of hypertension, diabetes mellitus, smoking status, and heart disease (including atrial fibrillation and coronary artery disease). Stroke severity was evaluated using the National Institutes of Health Stroke Scale (NIHSS) (24) at admission. Stroke subtype was classified according to Trial of Org 10,172 in Acute Stroke Treatment (TOAST) as large artery atherosclerosis (LAA), small artery occlusion (SAO), cardioembolism (CE), and other types (other cause [SOD] and undetermined cause [SUE]) (25). Mechanical thrombectomy was also recorded. Laboratory tests included neutrophil-to-lymphocyte ratio (NLR) (26), monocyte-to-lymphocyte ratio (27), monocyte-to-high density lipoprotein ratio (MHD) (28), and platelet-to-lymphocyte ratio (PLR) (29). Estimated glomerular filtration rate (eGFR) was calculated using the Chronic Kidney Disease Epidemiology Collaboration formula (30) and was included as a covariate in the multivariate logistic regression model as in previous studies (31), with a cutoff value of 60 mL/min/1.73 m^2^.

### 2.6 Statistical analysis

Descriptive analysis of the entire study population was performed. Categorical variables are presented as a number and percentage; normally distributed continuous variables are reported as mean and standard deviation, and data with a non-normal (skewed) distribution are presented as median and interquartile range. Differences between groups were evaluated with the chi-squared test or Fisher’s exact test for categorical variables and the Student’s *t*-test or Mann–Whitney U test for continuous variables.

To examine the association between IVT and early-onset PSD while minimizing potential bias and confounding effects, we used a logistic regression model with PSM, with a 1:1 nearest neighbor matching algorithm and caliper width of 0.2. The propensity score (PS) was generated based on the aforementioned clinical parameters that are known to influence PSD occurrence, and the degree of matching was assessed using standardized mean difference with a threshold <0.1 considered acceptable.

Outcomes of the 2 groups in the PS-matched cohort were compared using a 2-sided *t*-test, with estimated PSs used as weights. A weighted cohort was generated using a pairwise algorithm (PA) (32) and inverse probability of treatment weighting (IPTW) (33). The PA model Pairs individuals in the treatment and control groups based on PSs that estimate the probability of receiving the treatment, thereby creating matched confounding for unbiased comparisons (32). The IPTW model assigns weights to individuals based on their PSs, giving more weight to those with similar treatment propensities and adjusting for confounding (33); this method allowed a more accurate estimation of the effect of treatment on outcome. We calculated the *E*-value to determine the contribution of potential unmeasured confounding factors to the observed association between IVT and early-onset PSD (34). To further assess the stability of our results, subgroup and interaction analyses were conducted for age, sex, smoking, history of stroke, hypertension, diabetes mellitus, and heart disease.

Statistical analyses were performed using R version 4.3.1^1^ and Free Statistics software version 1.8 (35). Comparisons were performed with a 2-tailed test with a significance threshold of *p* < 0.05.

## 3 Results

### 3.1 Baseline characteristics of the study population

The characteristics of the 633 study participants are presented in Table 1. The average age was 63.9 ± 11.6 years and 40.9% were female. Patients in the IVT group were younger and had lower rates of hypertension, diabetes mellitus, history of stroke, and heart disease than those in the non-IVT group. For TOAST stroke subtypes, the IVT group had a lower prevalence of LAA and SAO and higher prevalence of CE and SOD+SUE; additionally, a larger proportion received mechanical thrombectomy and eGFR and NLR were higher compared with the non-IVT group. There were no differences in sex ratio, smoking status, or NIHSS score at admission between groups.

**Table 1:** Baseline characteristics of participants by intravenous thrombolysis administration.

Characteristic	Unmatched Patients			SMD	Propensity-Scoretsbolysississisti			SMD
	All patients	Non-IVT	IVT		All patients	Non-IVT	IVT	
	(*n* = 633)	(*n* = 513)	(*n* = 120)		(*n* = 233)	(*n* = 116)	(*n* = 116)	
Age (year), mean (ets)	63.9 ± 11.6	64.22 ± 11.6	62.68 ± 11.57	0.133	62.53 ± 12.2	62.6 ± 12.7	62.5 ± 11.7	0.007
Female, sex, *n* (%)	259 (40.9)	207 (40.4)	52 (43.3)	0.06	101 (43.5)	51 (44.0)	50 (43.1)	0.017
Smoker, *n* (%)	82 (13.0)	68 (13.3)	14 (11.7)	0.048	27 (11.6)	13 (11.2)	14 (12.1)	0.027
Hypertension, *n* (%)	448 (70.8)	372 (72.5)	76 (63.3)	0.198	146 (62.9)	72 (62.1)	74 (63.8)	0.036
Diabetes mellitus, *n* (%)	182 (28.8)	157 (30.6)	25 (20.8)	0.225	52 (22.4)	27 (23.3)	25 (21.6)	0.041
History of stroke, *n* (%)	107 (16.9)	94 (18.3)	13 (10.8)	0.213	27 (11.6)	14 (12.1)	13 (11.2)	0.027
Heart diseases, *n* (%)	47 (7.4)	33 (6.4)	14 (11.7)	0.183	26 (11.2)	15 (12.9)	11 (9.5)	0.109
NIHSS at admission, median (IQR)	2.0 (1.0, 5.0)	2.0 (1.0, 4.0)	3.5 (2.0, 6.0)	< 0.001	3.0 (1.0, 6.0)	3.0 (1.0, 8.0)	3.0 (2.0, 6.0)	0.017
TOAST subtype, *n* (%)				0.183				0.149
LAA	332 (52.4)	271 (52.8)	61 (50.8)		113 (48.7)	54 (46.6)	59 (50.9)	
SAO	208 (32.9)	173 (33.7)	35 (29.2)		69 (29.7)	34 (29.3)	35 (30.2)	
CE	46 (7.3)	34 (6.6)	12 (10.0)		25 (10.8)	15 (12.9)	10 (8.6)	
SOD+SUE	47 (7.4)	35 (6.8)	12 (10.0)		25 (10.8)	13 (11.2)	12 (10.3)	
Mechanical thrombectomy, *n* (%)	7 (1.1)	3 (0.6)	4 (3.3)	0.199	3 (1.3)	2 (1.7)	1 (0.9)	0.076
eGFR (mL/min/1.73 m^2^), mean73	96.7ean73	96.0ean73	99.3ean73	0.166	100.4 n73 m	101.36 (20.80)	99.35(19.85)	0.099
NLR, median (IQR)	2.5 (1.9, 3.7)	2.5 (1.9, 3.6)	2.8 (2.1, 4.4)	0.179	2.7 (2.0, 4.0)	3.36 (2.81)	3.46(2.49)	0.034
MLR, median (IQR)	0.3 (0.2, 0.4)	0.3 (0.2, 0.4)	0.3 (0.2, 0.4)	0.016	0.3 (0.2, 0.4)	0.34 (0.18)	0.35(0.17)	0.034
PLR, median (IQR)	124.3 (95.5, 159.0)	124.7 (95.7, 158.3)	118.7 (94.3, 160.0)	0.058	120.4 (94.3, 159.3)	134.42 (56.68)	130.47(57.76)	0.069
MHD, median (IQR)	0.5 (0.4, 0.7)	0.5 (0.4, 0.7)	0.5 (0.3, 0.7)	0.101	0.5 (0.4, 0.7)	0.55 (0.23)	0.55(0.28)	0.017

Continuous variables were expressed as mean Vataja, R.; Kainterquartile range. Categorical variables are expressed as frequencies (percentages). IVT, intravenous thrombolysis; SMD, standardized mean difference; NIHSS, National Institutes of Health Stroke Scale; TOAST, Trial of Org 10,172 in acute stroke treatment; LAA, large artery atherosclerosis; SAO, small artery occlusion; CE, cardioembolic; SOD, stroke of other causes; SUE, stroke of undetermined causes; eGFR, estimated glomerular filtration rate; NRL, neutrophil-to-lymphocyte ratio; MLR, monocyte-to-lymphocyte ratio; PRL, platelet-to-lymphocyte ratio; MHD, monocyte-to-HDL ratio.

**Figure 2:**
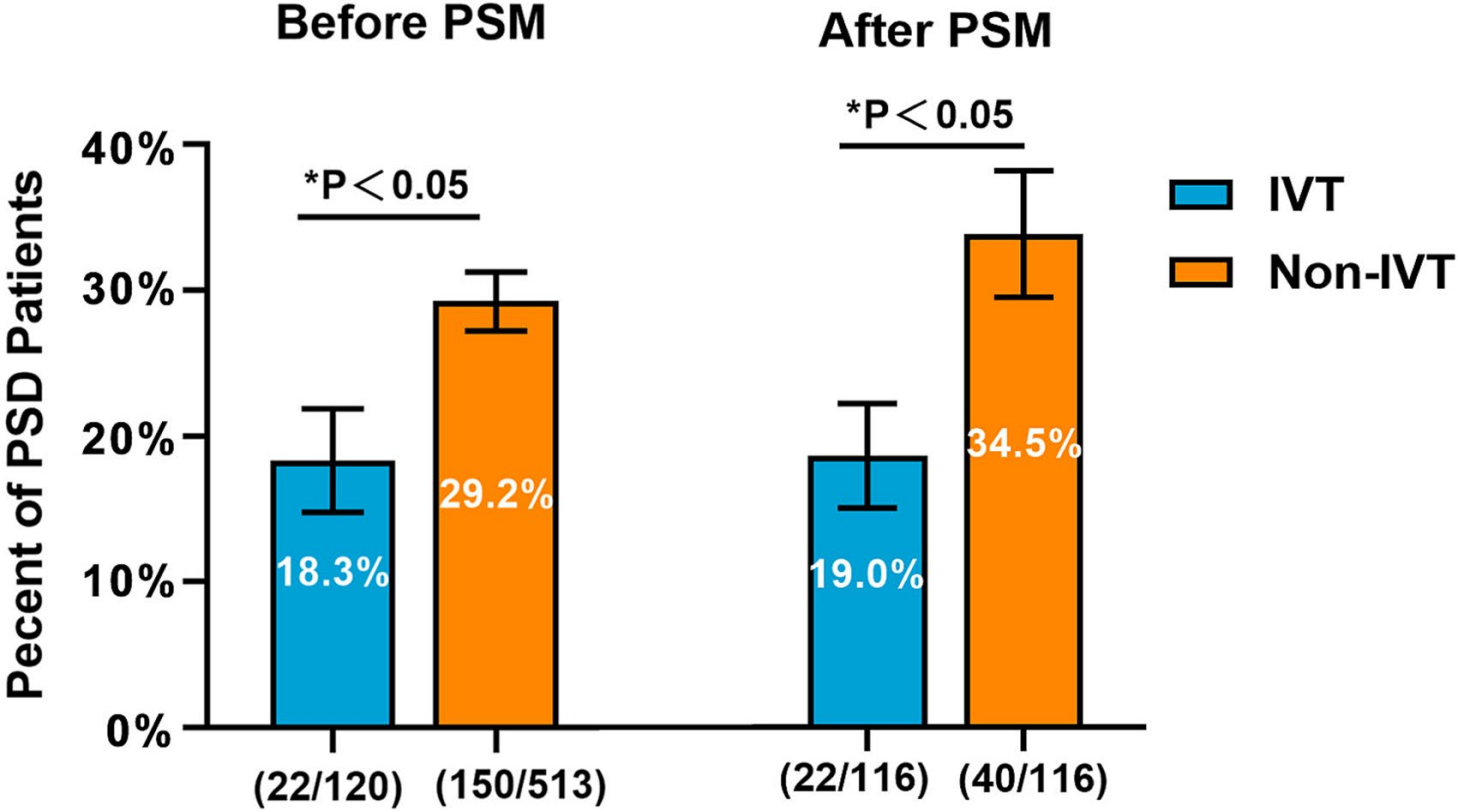
Percent of patients with or without PSD by intravenous thrombolysis administration. PSD, post-stroke depression; IVT, intravenous thrombolysis; PSM, propensity score method.

Before PSM, the prevalence of PSD was 18.3% (22/120) in patients with IVT and 29.2% (150/513) in those without IVT. After PSM, the baseline characteristics of the 2 groups were almost balanced, and the prevalence of PSD was 19.0% (22/116) and 34.5% (40/116), respectively. Treatment with IVT was associated with significantly lower rates of early-onset PSD (*p* < 0.05; Figure 2).

### 3.2 Outcomes in matched cohorts

IVT was associated with a significant reductions in the occurrence of early-onset PSD in both univariate and multivariate logistic regression analyses, after IPTW or using the PA model based on PS; odds ratios (ORs) ranged from 0.44 to 0.61 (all *p* < 0.05; Table 2). The *E*-value for the study population was 2.24; that is, unmeasured confounders would have to be more than 2 times more prevalent in the IVT group than in the non-IVT group to explain the observed association.

**Table 2:** Associations between IVT and PSD in the crude analysis, multivariable analysis, and propensity-score analyses.

Analysis	OR (95%)	*p*- value
Crude analysis tween IVT and	0.54 (0.33–0.9)	0.017
Multivariable analysis T and PSD in ^a^	0.48 (0.28–0.83)	0.008
With inverse probability weighting^b^	0.61 (0.37–0.99)	0.044
With matching^c^	0.44 (0.24–0.81)	0.008
With pairwise algorithmic^d^	0.5 (0.27–0.92)	0.026
Adjusted for propensity score^e^	0.52 (0.31–0.86)	0.012

IVT, intravenous thrombolysis; PSD, post-stroke depression; OR: odds ratio; CI, confidence interval. ^a^Shown is the odds ratio from the multivariable logistic regression model, with adjusted for all covariates in Table 1. ^b^Shown is the primary analysis with an odds ratio from the multivariable logistic regression model with the same strata and covariates with inverse probability weighting according to the propensity score. ^c^Shown is the odds ratio from a multivariable logistic regression model with the same strata and covariates with matching according to the propensity score. The analysis included 116 patients (116 who received thrombolysis administration and 116 who did not). ^d^Shown is the odds ratio from a multivariable logistic regression model with the same strata and covariates, with pairwise algorithmic according to the propensity score. ^e^Shown is the odds ratio from a multivariable logistic regression model with the same strata and covariates, with additional adjustment for the propensity score.

### 3.3 Subgroup analyses

**Figure 3:**
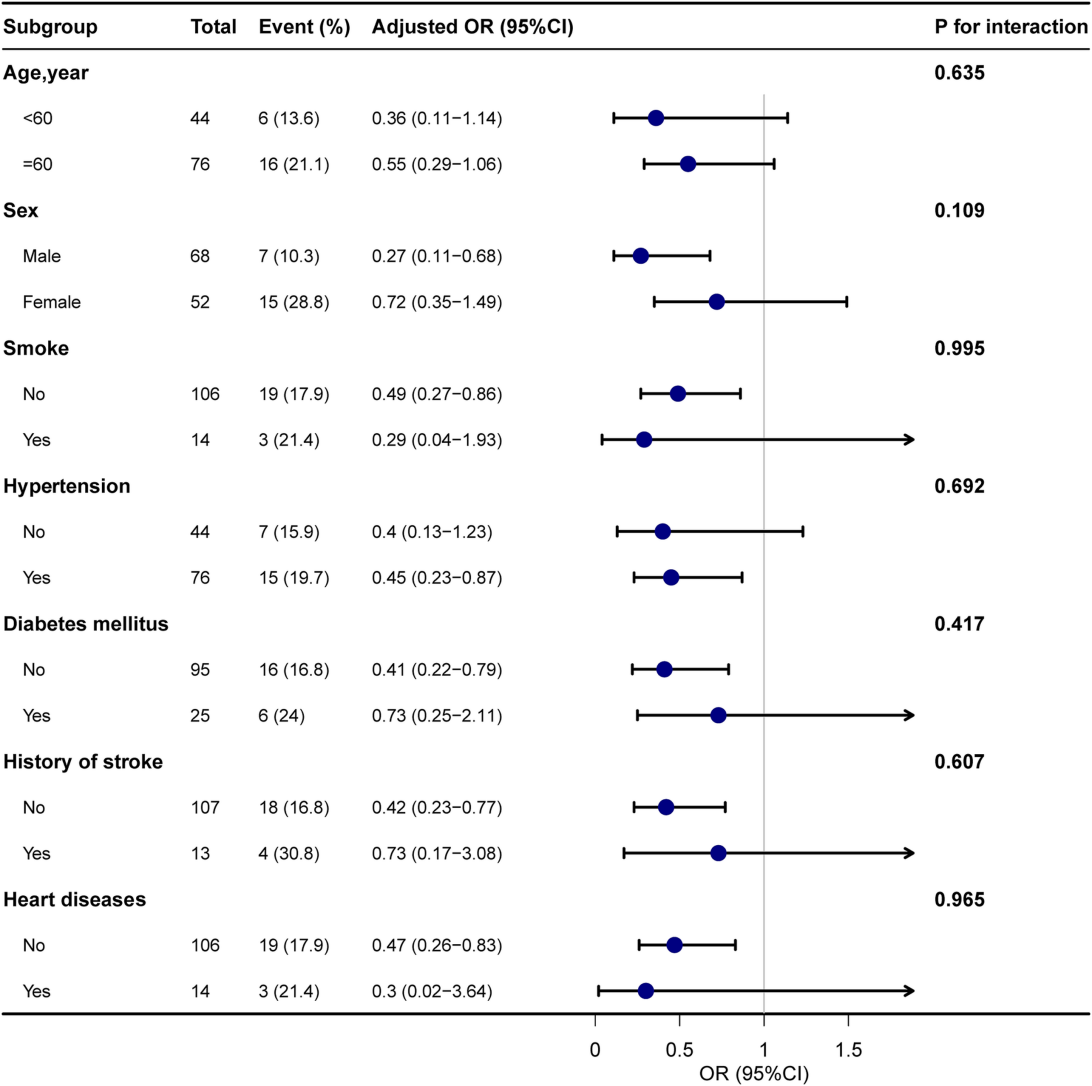
Forest plot shows ORs of PSD in subgroup analyses. Adjusted for all covariates in table1. PSD, post-stroke depression; OR, odds ratio; CI, confidence interval.

In subgroup analyses, there were no effects of interactions between age, sex, smoking status, hypertension, diabetes mellitus, history of stroke, or heart disease on the association between IVT and early-onset PSD using a fully adjusted model (Figure 3).

## 4 Discussion

This study investigated the relationship between IVT and early-onset PSD. We found that IVT decreased the occurrence of PSD in the 14–21 days after stroke by 52% compared with no IVT. The results remained robust after patients in the 2 groups were matched on baseline characteristics and in subgroup analyses of patients stratified by age and other factors known to influence PSD risk.

Of the 633 patients in the study population, 172 were diagnosed with early-onset PSD at a rate of 27.2%, similar to the overall rate of 29–43% reported in other studies (1–5). In the WAKE-UP trial post-mortem analysis, the prevalence of PSD was 42.9% with IVT vs. 52.7% without IVT, representing an absolute difference of 18.6% (17) compared with 37.3% in our work. However, there was limited laboratory testing in the study by Königsberg et al. Previous studies have shown that inflammatory indicators such as NLR (26), PLR (29), and MHD (28) are elevated in the peripheral blood of patients with PSD compared with non-depressed patients. Our study accounted for these variables. In another prospective study, rates of PSD were comparable between patients who received thrombolysis and those who did not at 3 and 12 months of follow-up. However, it is worth noting that the study sample comprised just 74 patients whereas in our study there were 116 patients in each group after PSM.

Treatment with intravenous alteplase led to an absolute risk reduction of PSD—as measured by HAMD17—at 14–21 days post stroke, consistent with the results of a previous randomized controlled trial (19). Previous studies have focused on the impact of thrombolysis on PSD at 90 days after stroke, but we examined its effect in the acute stage. After PSM, baseline characteristics including age, sex, hypertension, and NIHSS score at admission that are known predictors of PSD (3) were similar between IVT and non-IVT groups. The *E*-value of 2.24 provided further support that the association between IVT and PSD was robust to the effects of unknown confounders. In the comparison of the 116 matched pairs, the IVT group had fewer occurrences of PSD than the non-IVT group (*p* < 0.05) after controlling for PSD risk factors. This was also observed in subgroup analyses of patients stratified by age, sex, smoking, history of stroke, hypertension, diabetes mellitus, and heart disease. These findings suggest that IVT has a mitigating effect on early-onset PSD.

There are several possible mechanisms underlying the effects of IVT on early-onset PSD. First, the timely restoration of cerebral blood flow via thrombolysis can reduce the extent of neuronal damage, thereby protecting neural circuits involved in the regulation of emotion (36) such as the monoamine neurotransmitter system (37), which encompasses the cortical and limbic systems. Ischemic injury can directly injure monoaminergic neuron nuclei or their ascending projections, reducing monoamine neurotransmitter release. For example, damage to the basal ganglia results in dysfunction of emotional regulation, fatigue, and cognitive impairment (38). Thrombolysis may reduce the risk of PSD by alleviating brain damage after AIS. It may also affect biochemical and neurochemical processes that are induced after stroke and are implicated in depression; in addition to the disruption of neurotransmission, these include inflammation and oxidative stress (39).

The acute inflammatory response after stroke is reflected by elevated levels of central and peripheral proinflammatory cytokines and increases in NLR (26), PLR (29), and MHD (28) that lead to glucocorticoid release (40). Chronic inflammation can result in dysregulation of the hypothalamic–pituitary–adrenal axis and PSD. Inhibition of the NOD-like receptor protein 3 (NLRP3) inflammasome was shown to diminish depression-like behavior in rats following stroke (41). However, rtPA can induce the upregulation of genes encoding inflammatory factors such as NLR, thereby aggravating inflammation (42). We speculate that thrombolysis modulates these pathologic processes by restoring blood flow and reducing ischemic damage to alleviate depression symptoms while also improving functional outcomes and QoL after stroke (19). Additionally, patients with better recovery (less disability and improved physical functioning) after stroke may have a more positive mental state, potentially decreasing the risk of depression (43, 44, 45).

The impact of thrombolysis on depression should be assessed as part of a comprehensive stroke management plan that includes rehabilitation, psychological interventions, and long-term follow-up. For instance, initiating active antidepressant treatment immediately after a diagnosis of early PSD may reduce depression symptoms, promote functional recovery, and improve patients’ QoL.

### 4.1 Limitations

This study was not without limitations. First, patients with severe stroke experiencing disorders of consciousness or speech were excluded because their impairment prevented them from completing the HAMD. Consequently, the occurrence of PSD in patients with AIS may have been underestimated. Second, given that the study was hospital-based, it was difficult to assess depression scores prior to stroke onset and the potentially confounding effect of preexisting depression symptoms cannot be completely excluded. In future studies with larger cohorts, depression scores before and after stroke should be compared (46). Finally, large-scale multicenter studies are needed to confirm our findings and validate their applicability to clinical practice, and to elucidate the mechanisms by which thrombolysis mitigates early-onset PSD.

## 5 Conclusion

IVT treatment was associated with a 52% decrease in the occurrence of early-onset PSD 14–21 days after AIS compared with no IVT. These findings underscore the potential benefits of thrombolysis in preventing PSD and highlight the importance of an integrated approach to care in order to ensure optimal psychological outcomes and improve QoL in patients with stroke.

## Data Availability

The raw data supporting the conclusions of this article will be made available by the authors, without undue reservation.

## Footnotes

^1^http://www.R-project.org; The R Foundation, Vienna, Austria
